# Polymorphic Variation of Genes in the Fibrinolytic System and the Risk of Ovarian Cancer

**DOI:** 10.1371/journal.pone.0005918

**Published:** 2009-06-15

**Authors:** Yaakov Bentov, Theodore J. Brown, Mohammad R. Akbari, Robert Royer, Harvey Risch, Barry Rosen, John McLaughlin, Ping Sun, Shiyu Zhang, Steven A. Narod, Robert F. Casper

**Affiliations:** 1 Department of Obstetrics and Gynecology, University of Toronto, Toronto, Canada; 2 Samuel Lunenfeld Research Institute, Mount Sinai Hospital, Toronto, Canada; 3 Women's College Research Institute, Women's College Hospital, University of Toronto, Toronto, Canada; 4 Yale University, New Haven, Connecticut, United States of America; 5 Gynecologic Oncology, Princess Margaret Hospital, Toronto, Canada; Cincinnati Children's Research Foundation, United States of America

## Abstract

**Introduction:**

The etiology of ovarian cancer is largely unknown. One hypothesis is that the inefficient removal of the blood clots and fibrin products which are deposited in the vicinity of the ovary by retrograde menstruation might be associated with an increased risk of ovarian cancer. Several single nucleotide polymorphisms within genes which comprise the fibrinolytic system have been shown to have functional effects on the rate of blood clot degradation. These were considered to be candidate genes in the present study.

**Aim:**

We studied the genotype distributions of 12 functional SNPs of four genes (tPA, uPA PAI1 and TAFI) among 775 ovarian cancer cases and 889 controls.

**Results:**

No significant associations were seen between any of the ten SNPs and the risk of ovarian cancer as a whole, or in any histologic subgroup.

**Discussion:**

Germline known functional variants of genes in the fibrinolytic system are not associated with risk of ovarian cancer.

## Introduction

Although the cause of ovarian cancer is unknown, various risk factors appear to be related to reproduction, contraception and inflammation. Parity, breast-feeding, oral contraceptives and tubal ligation are all protective. In contrast, endometriosis and talc are among the few known risk factors. On the whole, these observations suggest that factors which diminish the number of ovulatory cycles are protective and factors that increase local inflammation may be carcinogenic.

Endometriosis is associated with a significantly increased risk of ovarian cancer [1], [2]. The prevalence of endometriosis in patients with epithelial ovarian cancer is 36% for clear cell carcinoma and 19% for endometrioid ovarian carcinoma. In one study, ovarian cancer was found in 5–10% of ovarian endometriotic lesions [3]. It is believed that retrograde menstruation is necessary for the development of endometriosis [4]. Tubal ligation and oral contraceptives prevent (or reduce) retrograde menstruation and both are associated with a reduction in the risk of ovarian cancser [5]–[8].

Despite the high prevalence of retrograde menstruation in up to 90% of women [9], [10], the prevalence of endometriosis is estimated to be in the range of 7–10% of women of reproductive age. One speculative explanation for this discrepancy is that an intact fibrinolytic process clears blood clots and endometrial cells from pelvic structures. We hypothesise that women with a defective fibrinolysis system may not remove blood clots effeciently and, as a result, this increases the time that endometrial cells in menstrual blood clots remain in contact with pelvic structures. These cells might possibly implant on the peritoneal or ovarian surface[11]. If this hypothesis is correct, there may be an association between defective fibrinolysis and the risk of ovarian cancer.

The fibrinolytic system comprises a family of proteins that includes two plasminogen activators (urokinase-type plasminogen activator (uPA) and tissue-type plasminogen activator (tPA)), the zymogen plasminogen, the active form plasmin, and inhibitor proteins like plasminogen activator inhibitor type 1 (PAI1) and thrombin-activated fibrinolysis inhibitor (TAFI).

The tPA is the primary mediator of local intravascular fibrinolysis. Genetic factors play a role in the variation of endothelial t-PA release [12]. The c.−7351C>T variant (rs2020918) within the enhancer region of the t-PA gene is strongly correlated with endothelial t-PA release rates. This SNP is associated with an increased risk of myocardial infarction [13].

Elevated levels of uPA promote tumor cell spread and metastasis and are associated with relatively poor prognosis [14]. A functional polymorphism of the *uPA* gene has been described. This is a substitution of C to T in the nucleotide sequence of exon 6 encoding the kringle domain resulting in Pro to Leu replacement at codon 141 (rs2227564).

PAI1 is a serine protease inhibitor that binds to both plasminogen activators, t-PA and u-PA, forming a stable complex that is cleared from the circulation by hepatic cells [15]. High levels of PAI1 are a common finding in ovarian cancer [16]. PAI1 over-expression is also associated with poor survival in ovarian cancer patients [17]–[19]. Furthermore, it has been suggested that PAI1 levels are important in the prognosis of breast and cervical cancers [20]–[22]. In cancer transplantation models, tumor growth, invasion, and angiogenesis are diminished in PAI1- deficient mice [23], [24]. Several single nucleotide polymorphisms (SNPs) in the PAI1 gene have been associated with a significant increase in PAI1 protein expression [25].

Thrombin-activated fibrinolysis inhibitor (TAFI) is a potent inhibitor of fibrinolysis that removes carboxy terminal–lysine residues from partially-degraded fibrin and decreases plasminogen binding [26]. *In vivo* animal studies demonstrate that inhibition of TAFI activity by carboxypeptidase increases thrombolysis [27]. Circulating levels of TAFI are strongly controlled by six polymorphic variations in the promoter and the 3'UTR region of the TAFI gene [28].

We propose that functional variants in these genes may be related to ovarian cancer risk. The objective of the present study was to determine if any of known functional polymorphisms of these four genes of the fibrinolytic system are associated with an increased risk of invasive ovarian cancer.

## Materials and Methods

### Study Population

Cases were ascertained through the Ontario Cancer Registry. All women newly diagnosed with invasive epithelial ovarian cancer in Ontario, Canada, from January 1995 to December 1999 were eligible. Of 1694 potentially eligible cases, 1016 women consented and provided blood samples for DNA testing. There were 775 women for whom both a DNA sample and sufficient clinical information were available and these are the subjects of the current study. Patients were categorized in four ethnic groups of Caucasian; French Canadian, East Asian and Indian (table 1).

**Table 1:** Subject characteristics of the ESCC cases and controls.

Variable		Cases	Controls	P-Value
Total Number, n		775	889	–
Age, Mean (range)		57.8 (26–79)	56.3 (28–94)	0.003
Ethnicity, n (%)	Caucasian	654 (84.4)	814 (91.5)	0.0002
	French-Canadian	77 (9.9)	65 (7.3)	
	East Asian	33 (4.3)	5 (0.6)	
	Indian	11 (1.4)	5 (0.6)	
Histology, n (%)	Serous	407(52.5)	–	–
	Endometrioid	176 (22.7)	–	
	Mucinouse	73 (9.4)	–	
	Clear Cell	49 (6.3)	–	
	Other	70(9.1)	–	

One thousand sixty-three controls were selected from healthy women who attended a screening clinic for well-women at the Women's College Hospital, Toronto, between 1996 and 2001. Of the women who were approached to participate in this study, approximately 80% agreed and provided a blood sample and completed a risk factor questionnaire. All study subjects provided informed consent for genetic testing. Controls had not been diagnosed with cancer. Study subjects were asked to provide details about their ethnic origins, including information about the place of birth of their four grandparents. 889 of these healthy women who were from the four ethnic groups of the cases were enrolled in this study.

We analyzed DNA samples from 1664 subjects, 775 cases with ovarian cancer and 889 controls. Patients known to carry a BRCA1 or BRCA2 mutation were excluded. Table 1 provides demographic information on the cases and controls.

### Genotyped Variants

Each DNA sample was checked for a total of 12 SNPs in the four candidate genes. All these 12 variants were shown to affect the expression or function of their related gene or protein [13], [25], [28]. These variants include rs2020918 (c.−7351C>T) from tPA gene; rs2227564 (Pro141Leu) from uPA gene; rs1799889, rs2227631, rs2227674 and rs6465787 from PAI1 gene; and rs1926447 (Thr347Ile), rs3581491, rs2146881, rs3742264 (Ala169Thr), rs1087 and rs34813434 from TAFI gene.

iPLEX chemistry on a MALDI-TOF MassARRAY system (Sequenom Inc., San Diego, CA, USA) was used for genotyping the 12 SNPs in eight reactions. The procedures were performed according to the manufacturer's standard protocol [29].

### Statistical methods

Deviations of genotype frequencies in the controls from those expected under Hardy Weinberg equilibrium (HWE) were assessed by χ^2^ tests (1 degree of freedom). All case-control comparisons were adjusted for age and ethnicity, using multivariate logistic regression and the adjusted P-values and odds ratios (OR) were reported. Given the number of comparisons in this study, a p-value of <0.01 was used as the criterion of statistical significance. Associations between ovarian cancer and SNP genotypes were measured in the study group as a whole and then in subgroups defined by histological type, age of diagnosis and family history. Family history was defined as one or more first- or second-degree relatives with breast cancer under age 50 or ovarian cancer.

## Results

Twelve SNPs, representing four genes were examined in 775 ovarian cancer cases and 889 controls. Two SNPS in TAF1 (rs1087 and rs34813434) were excluded because of call rates below 90%. The call rates for the other 10 SNPs were all in excess of 95% and all were in Hardy-Weinberg equilibrium among controls. The genotypes for these ten SNPs are shown in table 2.

**Table 2:** Genotypes of the 11 functional variants of 4 genes on the 775 cases and 889 controls.

Variant	Gene	MAF*	Cases, n (%)	Controls, n (%)	OR†	P-Value†
**rs2020918**	**tPA**	0.34				
C/C			532 (70.8)	647 (73.4)	1.00	1.00
C/T			206 (27.4)	219 (24.8)	0.95	0.60
T/T			13 (1.8)	16 (1.8)	1.07	0.37
**Pro141Leu**	**uPA**	0.25				
Pro/Pro			427(55.3)	496 (55.8)	1.00	1.00
Pro/Leu			300 (38.9)	340 (38.2)	1.00	0.97
Leu/Leu			45 (5.8)	53 (6.0)	1.00	0.97
**rs1799889**	**PAI1**	0.46				
4G4G			226 (29.3)	257 (28.9)	1.00	1.00
4G5G			372 (48.2)	440 (49.5)	0.96	0.71
5G5G			174 (22.5)	192 (21.6)	1.02	0.82
**rs2227631**	**PAI1**	0.41				
A/A			260 (33.9)	315 (35.5)	1.00	1.00
A/G			362 (47.3)	425 (47.9)	1.03	0.79
G/G			144 (18.8)	148 (16.6)	1.08	0.27
**rs2227674**	**PAI1**	0.21				
A/A			452 (59.7)	558 (63.0)	1.00	1.00
A/G			268 (35.4)	295 (33.3)	1.12	0.30
G/G			37 (4.9)	33 (3.7)	1.17	0.20
**rs6465787**	**PAI1**	0.01				
C/C			749 (97.0)	866 (97.4)	1.00	1.00
C/T			23 (3.0)	22 (2.5)	1.17	0.62
T/T			0 (0.0)	1 (0.1)	–	–
**Thr347Ile**	**TAFI**	0.30				
Thr/Thr			374 (48.6)	450 (50.6)	1.00	1.00
Thr/Ile			327 (42.5)	354 (39.8)	1.12	0.29
Ile/Ile			69 (8.9)	85 (9.6)	0.97	0.73
**rs35814191**	**TAFI**	0.34				
C/C			314 (43.0)	388 (43.8)	1.00	1.00
C/−			320 (43.8)	396 (44.7)	1.06	0.57
−/−			96 (13.2)	101 (11.5)	1.10	0.24
**rs2146881**	**TAFI**	0.26				
G/G			398 (55.0)	482 (54.6)	1.00	1.00
G/A			277 (38.3)	335 (37.9)	0.99	0.90
A/A			49 (6.7)	66 (7.5)	0.93	0.44
**Ala169Thr**	**TAFI**	0.32				
Ala/Ala			346(46.3)	392 (44.3)	1.00	1.00
Ala/Thr			324 (43.4)	405 (45.8)	0.96	0.68
Thr/Thr			77 (10.3)	88 (9.9)	1.00	0.96

* Minor Allele Frequency.

† P-value and odds ratio (OR) are adjusted for age and ethnicity using multivariate logistic regression analysis.

For none of the 10 studied SNPs was the distribution of genotypes significantly different between the cases and controls (table 2). Sub-division of cases based on the age at diagnosis or histological type did not yield any significant association (data not shown).

## Discussion

We hypothesized that an inherited defect in the fibrinolytic pathway could lead to an increased duration of exposure of the ovaries to blood clots containing epithelial cells originating from the Mullerian tract deposited by retrograde menses, and thereby increase the risk of ovarian cancer. This hypothesis was based on the known protective effect of tubal ligation and oral contraceptives against ovarian cancer, which prevent or reduce retrograde menstruation. In addition, previous research has demonstrated an increased activity of inhibitors of the fibrinolytic system in ovarian cancer patients. Despite the large number of samples and SNPs examined, the results of the present study were negative. The ovarian cancers that have been most strongly associated with endometriosis are the clear-cell and endometriod subtypes. Notably, in neither of these subgoups was an association found.

Increased expression of several fibrinolytic modulators has been associated with increased risk for cancer development and poor prognosis [14]–[24]. Specifically, PAI1 was shown to promote tumor growth in a dose dependent and stage-dependent manner [30]. Moreover, the SNP rs1799889 in the promoter of the PAI1 gene was shown by 37 separate studies to poses a significant allelic dose-dependent correlation between the 4G allele and increased PAI1 protein level in vivo. The 4G/4G homozygotes have the highest levels of circulating PAI1 [26].

Sternlicht et al [25] detected an association between PAI1 levels and overall survival from breast cancer in a study of 2,539 cases and 1,832 controls. However, the PAI1 4G/5G SNP was not associated with breast cancer incidence, clinical outcome or PAI1 expression. These authors concluded that cancer–associated signals are more important than germline genetic variability in determining the expression of PAI1 in breast cancer patients. In our study, the 4G/4G, 4G/5G and 5G/5G genotypic distributions were similar in the cases and controls.

We included all the SNPs of genes of the fibrinolytic system that were found to be associated with a change in either the concentration of the protein or its function.

We failed to detect any significant association between fibrinolysis gene polymorphisms and the incidence of ovarian cancer in any histological subtype. If the fibrinolytic pathway is involved in ovarian cancer, the risk does not appear to be influenced by functional polymorphisms in the key genes. However, given the previous studies, which report a possible role for these enzymes in the initiation or progression of cancer, it may be that variation in the expression of the proteins in the fibrinolytic system remains relevant for ovarian carcinogenesis.
